# Comparison between viral vector and mRNA based COVID-19 vaccination in prevalence and severity of regional immune reactions, and ^18^F-FDG PET/CT features

**DOI:** 10.22038/AOJNMB.2022.63110.1443

**Published:** 2023

**Authors:** Narjess Ayati, Scott Evans, S. Rasoul Zakavi, Simon M. Gruenewald

**Affiliations:** 1 Department of Nuclear Medicine, Ultrasound & PET, Westmead Hospital, NSW, Australia; 2 Nuclear Medicine Research Center, Mashhad University of Medical Sciences, Mashhad, Iran

**Keywords:** BioNTech/Pfizer, COVID-19, Vaccination, ^ 18^F-FDG PET/CT, mRNA vaccine

## Abstract

**Objective(s):**

The coronavirus pandemic caused by SARS-CoV-2 commenced in late 2019, and global wide vaccination appears to be the only reasonable solution to fight this dreadful virus. There are two main types of COVID-19 immunization using viral vector and mRNA-based vaccines. However, the impact of each of type on ^18^F-FDG PET/CT needs to be accurately assessed. This study aimed to compare the ^18^F-FDG PET/CT features of these two types of COVID-19 vaccines.

**Methods:**

A total of 188 patients referred for ^18^F-FDG PET/CT with a recent history of either BioNTech/Pfizer or AstraZeneca COVID-19 vaccination, and a control group of 40 patients with no history of any type of recent vaccination, were included in the study. ^18^F-FDG PET/CT studies of vaccinated patients assessed for injection site uptake and regional nodal and systemic reactions post vaccination. The data were compared to the control group and to the contralateral side for each patient. The findings were compared between patients who received Pfizer and AstraZeneca vaccines.

**Results:**

^18^F-FDG PET/CT was semiquantitatively positive in 50.5% of the studied population for vaccine-related features. The ipsilateral axillary and infra- and supraclavicular lymph nodes were significantly larger in size and exhibited higher metabolic activity compared to the contralateral lymph nodes after both types of vaccination. The prevalence of regional nodal reactions post Pfizer and AstraZeneca vaccination was 39% and 17.9% on visual, and 61% and 47.6% on semiquantitative assessments, respectively. Patients receiving the Pfizer vaccine exhibited higher metabolic activity in the ipsilateral regional lymph nodes (p<0.05). No significant difference in the intensity of regional nodal reaction post vaccination was noted between the first four weeks.

**Conclusion:**

Significant local and regional nodal reactions are observed after both viral vector and mRNA COVID-19 vaccination with a tendency to extend toward the infra- and supraclavicular nodal stations but not to the pulmonary hilum. The greater intensity and extension of the nodal reaction after Pfizer vaccination suggests a higher possibility of false-positive results on ^18^F-FDG PET/CT studies using mRNA vaccination technology.

## Introduction

 The coronavirus pandemic caused by SARS-CoV-2 commenced in late 2019 with over 220 million confirmed cases and more than 4,600,000 reported related deaths as of the 18^th^ of September 2021 according to the World Health Organization (1). To fight this dreadful pandemic of COVID-19 (Coronavirus Disease 2019), the only reasonable solution is broad vaccination coverage. There are two main types of COVID-19 immunization using adenovirus DNA vectors and messenger ribonucleic acid (mRNA) vaccines.

 Although conventional vaccines have made a

great contribution in saving lives for over a century (2), the first successful animal study of mRNA vaccine application which was published in 1990 (3), showed its promising features over conventional vaccination technology, including safety (as a noninfectious, nonintegrating platform with capacity of downregulation of its inherent immunogenicity) (4-6), stability, efficacy, and the capacity of low-cost rapid vaccine development (7). In brief, adenovirus DNA vectors are carriers of genetic information for the SARS-COV-2 spike glycoprotein, whereas messenger RNA (mRNA) directly triggers the synthesis of the viral spike protein in receivers. The former includes AstraZeneca, Johnson & Johnson, and Sputnik, and the latter includes BioNTech/Pfizer and Moderna vaccines (8-9).

 It is well known that any inflammatory process (including vaccination-related immune reaction) may have an impact on ^18^F-FDG PET/CT imaging, resulting in false-positive findings (10). This not only involves the regional nodal region (11), but according to some previous reports, it may result in generalised immune reaction (10). Recently, regional and systemic immune reactions and subsequent ^18^F-FDG PET/CT findings post COVID-19 vaccination have been reported (12). However, as the mechanisms of these two types of COVID-19 vaccines are completely different, the provoked immune reaction and subsequently the prevalence, severity and persistence of ^18^F-FDG PET/CT findings may be quite different.

 Given that the world has entered a phase of massive immunization against COVID-19, the potential ^18^F-FDG PET/CT pitfalls post vaccination need to be precisely assessed. This study aimed to compare the PET features of these two types of COVID-19 vaccination.

## Methods

***Patients***

 A total of 810 consecutive patients who were referred to Westmead Hospital (NSW, Australia) between the 15^th^ of March and the 15^th^ of August 2021 for the ^18^F-FDG PET/CT study with a history of either a first or second dose of AstraZeneca (Vaxzevria) or BioNTech/Pfizer (Comirnaty) vaccination within the last 30 days were included in the study.

**Figure1:**
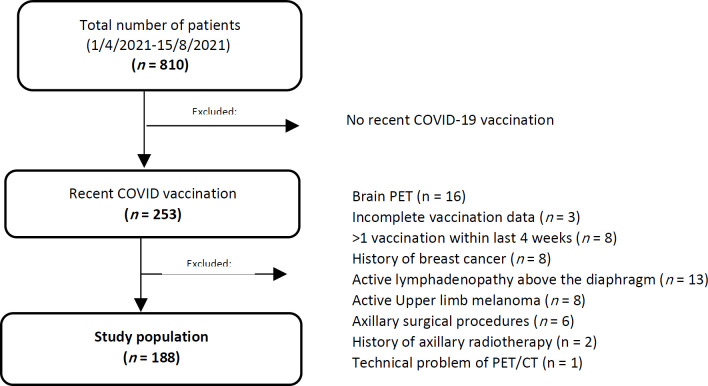
Flowchart of inclusion and exclusion criteria

**Figure 2:**
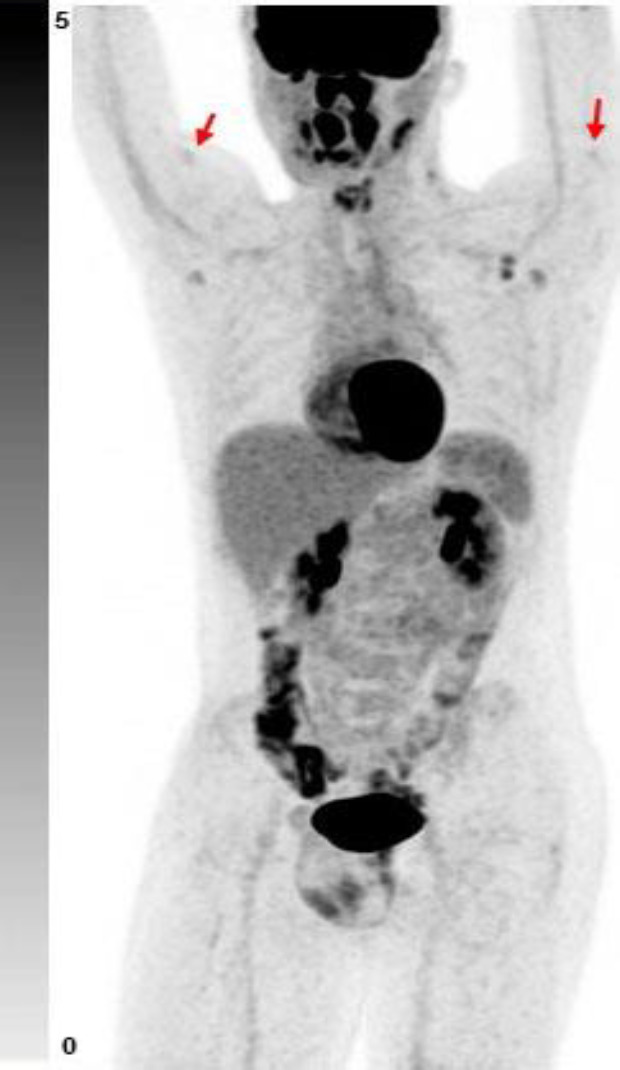
A 26-year-old gentleman with post-transplant lymphoproliferative disease presents for post chemotherapy response assessment. The patient had history of flu and Pfizer (2^nd^ dose) vaccination injected to the right and left arms 7 and 21 days before the PET scan respectively. Bilateral metabolically active axillary lymph nodes are seen. While the Pfizer vaccination was 14 days earlier than flu vaccination, the nodal reaction on the left side is more intense compared to the contralateral side. The left axillary lymph nodes measure up to 10mm in short axis diameter with SUV_max_ up to 3.8, and the right axillary lymph nodes measure up to 8mm with SUV_max_ up to 2.3. Bilateral mild deltoid uptake (SUV_max_: 1.3 right, 1.4 left) within the injection sites (**red arrows**) is also seen. Having more than one type of vaccination within the last month, the patient was excluded from the study

 Patients with a history of breast cancer, active upper limb melanoma, lymphoma with active lymphadenopathy above the diaphragm, history of nodal dissection or radiotherapy in either axillae, any other axillary surgical procedure (including lymph node biopsy) within the last two years, other oncologic disease with a high possibility of axillary involvement, more than one type of vaccination within the last 30 days and incomplete clinical or imaging data (e.g. brain imaging) were excluded from the study (Figure 1). The study was approved by the Health Human Research Ethics Committee (Approval number # 2107-04). Figure 2 shows an interesting case who was excluded from the study due to receiving two types of vaccination within the last 30 days prior to ^18^F-FDG PET/CT (Figure 2).

 Demographic data were collected. In addition, vaccine-related information (type and date of vaccination, site of injection and systemic symptoms post vaccination); disease-related information, including PET/CT indication and patients’ immune status; and past medical history of any diagnostic or therapeutic procedures involving the upper limbs or axillae were recorded (Table 1). The patients were considered immune compromised if they had any of the following conditions: current systemic corticosteroid treatment, history of chemotherapy within the last three months, immunotherapy within the last 6 months or active lymphoproliferative disease.

**Table 1:** General Characteristics of Patients, PET indication and vaccination data

**Parameter**	**Characteristic**	**Data**
**Age (year)**	Mean ± SD	67.2 ± 12.8
**Sex**	Female	83 (44.1)
	Male	105 (55.9)
**FDG PET/CT indication**	Non-oncology	19 (10.1)
	Oncology	169 (89.9)
**Immune status**	Immune compromised	57 (30.5)
	Immune competent	130 (69.5)
**Type of vaccine**	**AstraZeneca**	
	First dose	92 (48.9)
	Second dose	53 (28.2)
	**Pfizer**	
	First dose	23 (12.2)
	Second dose	20 (10.6)
**Injected arm**	Right	48 (25.5)
	Left	140 (74.5)
**Time interval between vaccination and PET (days)**	Mean ± SD	16.2 ± 9.0
**Vaccine-related systemic symptoms**	No	123 (83.7)
	Yes	24 (16.3)

Qualitative data are number followed by percentage (n=); continuous data are mean ± SD

***Control Group***

 Forty consecutive patients without a history of any type of vaccination within the last 3 months were selected as the control arm of the study. Given that most of the vaccinated patients received their injection in the left arm, for the control group, the left side was considered ipsilateral side, and the right side was considered the contralateral side in comparisons.

***Imaging Protocol***

 All patients fasted for at least 6 hours before the ^18^F-FDG PET studies. Blood glucose levels were checked before ^18^F-FDG injection. If the blood sugar level was less than 10 mmol/L, we proceeded with the study. Patients with blood sugars greater than 10 mmol/L were assessed 

on a case-by-case basis mostly rebooked after endocrinologist review. The ^18^F-FDG dose was in the range of 180–450 MBq and was determined according to the patient’s body mass index. The uptake time was 60-70 minutes for all patients. The standard imaging range for the PET/CT scans was midbrain to proximal femora. The imaging field was extended according to the clinical indications such as patients with melanoma or sarcoma.

 All imaging was performed on a Siemen Biograph mCT 128 slice PET/CT scanner. A low- dose CT scan was performed for attenuation correction and anatomic registration. Emission scans were performed for 2 minutes per bed position and reconstructed with Siemens True-X

 (ultraHD) time of flight reconstruction.

***Image Analysis***

 Images were analyzed by 2 nuclear medicine specialists on a computer display using a dedicated Siemens syngo via Client 5.2 software. 

 The maximum SUV normalized by body mass index (SUV_max_) was determined by the software within the region of interest (ROI) drawn at the site of vaccination on the deltoid muscles and overlying skin. A similar ROI was drawn on the contralateral side. The ipsilateral axillary lymph node with the highest metabolic activity on each side was detected at level I. If there was a detectable lymph node at axillary level II or III and, infra- or supraclavicular or cervical nodal stations, the size, shape and metabolic activity were recorded and compared with the contralateral side. If no lymph node was detectable in the contralateral area, a ROI was drawn in the background in that region. 

 Metabolic activity of bilateral pulmonary hilar regions was also recorded only if there was no evidence of lung malignancy, granulomatous disease or known mediastinal or pulmonary hilar lymphadenopathy and no history of mediastinal radiotherapy or recent respiratory infectious process. The SUV_max_ of the liver, spleen and bone marrow (lumbar vertebral ROI) were also calculated.

***Image Interpretation***

 The images were assessed visually and categorized into three categories of positive, negative, or retrospectively positive for both the vaccination site and regional (axillary) lymph nodes. In semiquantitative assessment, the result was considered positive if the ipsilateral to the contralateral count ratio was equal to or greater than 1.5. This comparison was applied for the injection site and regional nodal stations (including axilla, supra and infraclavicular, cervical and pulmonary hilar regions). The scan was positive overall for vaccination signs if visual or semiquantitative assessment was positive at either the injection site or nodal stations. 

Systemic immune reaction was assessed by calculating the ratio of the background metabolic activity of the spleen to the liver and the bone marrow to the liver.

***Statistical Analysis***

 Frequency statistics were obtained using frequency tables and descriptive analysis was done with SPSS software (version 26.0; SPSS Inc.). Quantitative variables were compared between different groups using independent t test t. Chi-square and McNemar tests were used for comparison of nominal variables among independent and dependent groups, respectively. P values of less than 0.05 were considered significant in all comparisons.

## Results

 In total, 253 patients with recent COVID-19 vaccination were identified, of whom 188 patients (105 male, 83 female) with a mean age of 67.2±12.8 years and history of recent COVID-19 immunization, including 43 Pfizer and 145 AstraZeneca vaccinations, and a control group of 40 patients (24 male, 16 female) with a mean age of 60.6±14.9 years and no history of any type of vaccination within the last three months were included in the study. The mean time interval between vaccination and PET imaging was 16.2±9.0 days and it ranged between 1 to 30 days (Median=16.5). The general characteristics of the patients are detailed in Table 1.

 There was no significant difference between the two groups in terms of age, ^18^F-FDG dose, blood glucose level, uptake time or metabolic activity of the liver, spleen, bone marrow and mediastinum (P>0.05). The metabolic activity of level I ipsilateral axillary lymph nodes was significantly higher in vaccinated patients than in the nonvaccinated group (P<0.001). Moreover, the SUV_max_ of ipsilateral to contralateral axillary (level I) lymph nodes was remarkably higher in vaccinated patients (P<0.001). In contrast, the SUV_max_ in levels II-III of axillary, supra/infraclavicular and pulmonary hilar lymph nodes showed no significant difference between vaccinated and nonvaccinated patients (Table 2).

**Table 2:** Comparison of PET parameters and metabolic activity of target organs and regional lymph nodes between vaccinated and non-vaccinated patients

**Parameter **		**Non-vaccinated (n=50)**	**Vaccinated (n=188)**	**P value**
**Age (year)**		65.6 ± 14.9	67.2 ± 12.8	0.07
^18^ **F- FDG dose (MBq)**		228.4 ± 48.3	237.1 ± 44.1	0.27
**Uptake time (min)**		64.6 ± 3.7	63.6 ± 3.5	0.09
**Blood Glucose (mmol/L)**		5.9 ± 1.3	5.7 ± 1.6	0.64
**Mediastinal Blood pool SUV**_max_		2.5 ± 0.5	2.5 ± 0.5	0.94
**Liver SUV**_max_		3.6 ± 0.6	3.6 ± 0.7	0.64
**Spleen SUV**_max_		2.7 ± 0.5	2.7 ± 0.5	0.71
**Axillary level I LN SUV**_max_				
	**Ipsilateral**	0.84 ± 0.35	1.35 ± 1.23	<0.001
	**Contralateral**	0.85 ± 0.37	0.74 ± 0.4	0.11
	**Ipsilateral/Contralateral**	1.03 ± 0.27	1.97 ± 1.79	<0.001
**Axillary level II/III LN SUV**_max_				
	**Ipsilateral**	1.07 ± 0.29	1.56 ± 1.12	0.33
	**Contralateral**	0.86 ± 0.21	0.73 ± 0.26	0.32
	**Ipsilateral/Contralateral**	1.24 ± 0.15	2.36 ± 2.03	0.22
**Supra/Infra-clavicular LN SUV**_max_				
	**Ipsilateral**	1.18 ± 0.04	1.46 ± 0.86	0.64
	**Contralateral**	0.85 ± 0.09	0.73 ± 0.28	0.58
	**Ipsilateral/Contralateral**	1.39 ± 0.19	2.1 ± 1.3	0.45
**Pulmonary hilar LN SUV**_max_				
	**Ipsilateral**	2.5 ± 0.46	2.6 ± 0.55	0.43
	**Contralateral**	2.5 ± 0.46	2.6 ± 0.59	0.53
	**Ipsilateral/Contralateral**	1.0 ± 0.11	1.0 ± 0.13	0.74

LN: lymph node; Continuous data are mean ± SD

 Overall, the PET images were visually positive (within either of the injection site or regional nodal stations) in 64 patients (34.4%) with 24 more patients (12.4%) retrospectively marked as positive. However, if deltoid activity was excluded, 42 patients (22.6%) had visually positive scans. On semiquantitative assessment, 93 vaccinated patients (50.5%) were categorized as positive. Bilateral nodal reaction was observed only in 3 patients (1.6%). 

 The frequency of developing vaccine-related systemic symptoms was significantly different between patients with (53.8%) and without (12.2%) extension of hypermetabolic nodal reaction toward level II and III axillary and infra-supraclavicular nodal stations (P=0.001). 

 On semiquantitative assessment, the SUV_max_ over the injection site was greater than that over the contralateral region (P<0.001). Similarly, both the size and metabolic activity of the lymph nodes were significantly higher in the ipsilateral axillary (level I-III) and infra- and supraclavicular regions (P<0.001) than in the contralateral side. However, this hyper-metabolism did not extend further to include pulmonary hilar lymph nodes (P=0.69).

 The mean age of the patients vaccinated with Pfizer was lower than that of patients who were vaccinated with AstraZeneca (54.1±14.9 versus 71.0±9.0 years, P<0.001). The mean blood glucose, mean dose of ^18^F-FDG and mean uptake time did not differ between the two groups (P > 0.05). The mean ipsilateral to contralateral deltoid SUV_max_ ratio was 1.3±0.48 in patients vaccinated with Pfizer while it was 1.4±0.70 in patients vaccinated with AstraZeneca (P=0.2). The prevalence of vaccine-related nodal reactions was more than twofold higher post Pfizer (39%) than after AstraZeneca vaccination (17.9%) on visual assessment (P=0.01). On semiquantitative assessment, the prevalence of ipsilateral hypermetabolic nodal reaction was 61% and 47.6% after Pfizer and AstraZeneca vaccines respectively which was not significantly different (P=0.1). The patients who received the Pfizer vaccine had significantly higher SUV_max_ in ipsilateral axillary (level I-III) and infra- and supraclavicular lymph nodes compared to patients vaccinated with AstraZeneca (Table 3). 

 Additionally, the frequencies of extended nodal reactions to the infra- and supraclavicular nodal stations were 17.1% and 2.1% after Pfizer and Astrazeneca vaccination, respectively (P<0.001). However, the metabolic activity of the spleen and bone marrow, and the ratio of metabolic activity of the spleen to the liver and bone marrow to the liver were not significantly different between patents that received either of these two types of vaccination (Table 3).

**Table 3:** Comparison of visual ^18^F-FDG PET/CT features and semi-quantitative parameters between patients received AstraZeneca versus Pfizer vaccination

**Parameter**	**Viral vector vaccination** **(AstraZeneca, n = 144)**		**mRNA vaccination** ** (Pfizer, n = 43)**	**P value**
**Visual assessment**				
**Positive FDG PET (any region)**		44 (30.3)	20 (48.8)	0.07
**Injection site (Deltoid uptake)**		31 (21.5)	7 (16.3)	0.53
**Regional nodal activation (any LN station)**		26 (17.9)	16 (39)	0.01
**Hypermetabolic axillary level I LN**		23 (15.9)	15 (36.6)	0.013
**Hypermetabolic axillary level II-III LN**		6 (4.2)	11 (26.8)	<0.001
**Hypermetabolic supra/infraclavicular LN**		3 (2.1)	7 (17.1)	<0.001
**Bone Marrow activation**		32 (22.7)	14 (34.1)	0.15
**Semi-quantitative assessment**				
**Regional nodal activation (any LN station)**		68(47.6)	25(61)	0.10
**Axillary level I LN SUV**_max_				
**Ipsilateral**		1.1 ± 0.73	2.0 ± 2.0	0.009
**Contralateral**		0.72 ± 0.28	0.78 ± 0.68	0.57
**Ipsilateral/Contralateral**		1.7 ± 1.1	2.9 ± 2.9	0.01
**Axillary level II/III LN SUV**_max_				
**Ipsilateral**		1.2 ± 0.56	2.4 ± 1.6	0.004
**Contralateral**		0.74 ± 0.26	0.71 ± 0.27	0.70
**Ipsilateral/Contralateral**		1.74 ± 0.83	3.8 ± 3.0	0.01
**Supra/Infra-clavicular LN SUV**_max_				
**Ipsilateral**		1.2 ± 0.61	1.8 ± 1.0	0.08
**Contralateral**		0.76 ± 0.28	0.67 ± 0.28	0.40
**Ipsilateral/Contralateral**		1.7 ± 0.93	2.9 ± 1.7	0.02
**Pulmonary hilar LN SUV**_max_				
**Ipsilateral**		2.6 ± 0.55	2.4 ± 0.50	0.06
**Contralateral**		2.6 ± 0.6	2.4 ± 0.50	0.052
**Ipsilateral/Contralateral**		1.0 ± 0.13	1.0 ± 0.16	0.92
**Injection site (Deltoid muscle)**				
**Ipsilateral/Contralateral**		1.4 ± 0.70	1.3 ± 0.48	0.20
**Liver SUV**_max_		3.7 ± 0.66	3.6 ± 0.65	0.59
**Spleen SUV**_max_		2.7 ± 0.5	2.6 ± 0.40	0.20
**Bone marrow SUV**_max_		2.6 ± 0.6	2.7 ± 0.6	0.32

LN: lymph node; Qualitative data are number followed by percentage; Continuous data are mean ± SD

**Figure 3:**
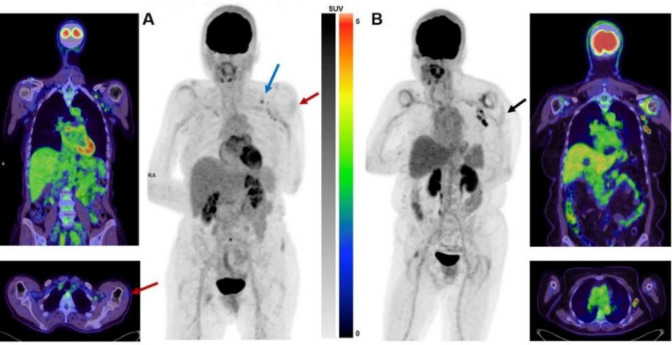
(**A**) A 62-year-old female with a new sacral nerve root enhancing lesion on MRI presents for further characterisation. There is a cluster of mild to moderately avid small left axillary lymph nodes at level1 (SUV_max_=3.1) and level 2 (SUV_max_=2.7) as well as left infraclavicular lymph node (SUV_max_=3.6) (**Blue arrow**). Mild activity is also seen in the superficial left deltoid muscle (SUV_max_=1.4) (**Red arrows**). The patient had Pfizer vaccination 6 days prior to PET scan injected to the left arm. (**B**) 67-year-old female with a poorly defined fat stranding in the right perinephric space suspicious for sarcoma presents for assessment. Multiple enlarged, intensely avid left axillary lymph nodes are noted (SUV_max_ up to 8.2). Low-grade activity is seen at the superficial aspect of the left deltoid (SUV_max_=1.5) (**Black arrow**). She had history of second dose Pfizer vaccination 5 days prior to PET scan

Figure 3 shows intensity and extension of nodal reaction in two patients received Pfizer vaccines.

**Figure 4:**
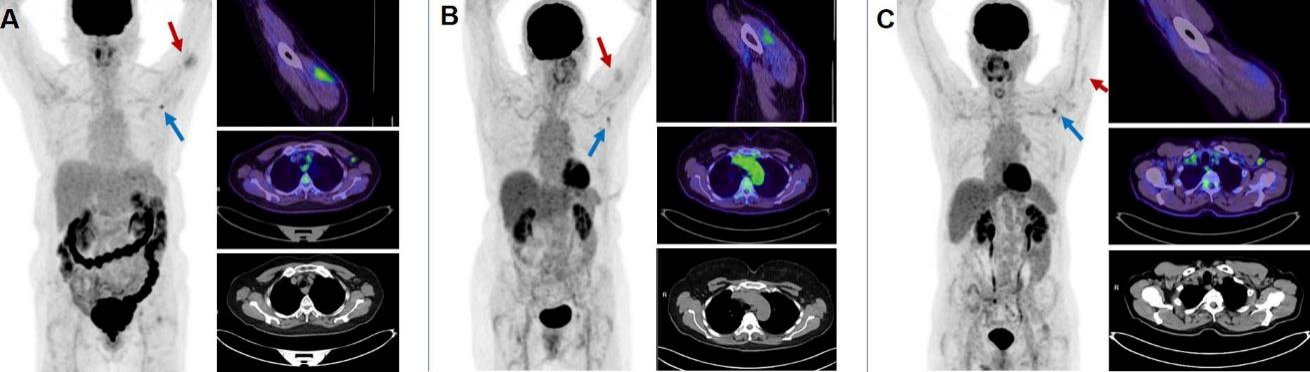
Vaccine-related ^18^F-FDG PET/CT features on day one (**A**), seven (**B**) and twenty-eight (**C**) after AstraZeneca vaccination. As seen on the image, the deltoid uptake reduces over time, however, the ipsilateral axillary nodal uptake remains stable over the first four weeks post vaccination. (**A**)A 60-year-old female with adenocarcinoma of the cervix 6-month post completion of brachytherapy presents for restaging. There was a minor increase in uptake through the musculature of the left upper arm laterally (SUV_max_=2.9) (**red arrow**) as well as in a mildly enlarged (11 mm) left axillary lymph node (SUV_max_=3.7) (**blue arrow**), consistent with recent COVID vaccination. She had received first dose of AstraZeneca one day prior to the study. (**B**)A 70-year-old female with right lower lobe ground glass changes for assessment. Two hypermetabolic left sided axillary lymph nodes (SUV_max_=3.4) (**blue arrow**) and increased posterior left deltoid uptake (SUV_max_=2.0) (**red arrow**) are related to AstraZeneca vaccination 7 days prior to imaging. (**C**)A 68-year-old male with a background history of follicular lymphoma, who recently has presented with multiple pulmonary nodules, for further evaluation. Few non-enlarged tracer avid lymph nodes in the left axilla (**blue arrow**) were noted (SUV_max_ up to 4.3). Mild FDG activity in the left deltoid muscle (**red arrow**) corresponds to the injection site of recent COVID vaccine. The patient had AstraZeneca vaccination 28 days prior to imaging

 The patients were categorized into four groups based on the time interval between their COVID-19 vaccination and PET study. ^18^F-FDG uptake in the injection site (deltoid muscle) was higher in the first week and significantly decreased in the following weeks (P=0.001). However, no statistically significant difference was noted between the 4 groups in metabolic activity of ipsilateral axillary (level I, II & III) or supra-/infraclavicular lymph nodes after vaccination. Similarly, the ipsilateral to contralateral SUV_max_ of lymph nodes were similar between the four groups (P>0.05) at all levels. The findings were analyzed for each type of vaccine separately (Table 4), and similar results were achieved in both groups (Figure 4).

**Table4:** (**A**): Quantitative positivity ratio as well as ipsilateral to contralateral SUV_max_ ratio according to time interval between Pfizer vaccination and PET

**Ipsilateral/contralateral(SUV**_max_ **) **	**1st Week**	**2nd Week**	**3rd Week**	**4th Week**	**P value**
**Quantitative positivity**	1.7±0.44	1.6±0.5	1.3±0.51	1.5±0.54	0.34
**Axilla (level I) **	3.4±3.6	2.1±1.1	1.8±2.0	4.2±4.0	0.32
**Axilla (level II)**	4.5±3.6	3.6±3.5	2.3±1.3	3.9	0.7
**Supra/Infraclavicular **	3.0 ±1.8	4.7±3.2	1.5±0.5	2.2±0.7	0.32
**Deltoid **	1.7±0.5	1.2±0.2	1.0±0.19	1.0±0.10	0.001

**Table4:** (**B**): Quantitative positivity ratio as well as ipsilateral to contralateral SUV_max_ ratio according to time interval between AstraZeneca vaccination and PET

**Ipsilateral/contralateral** **(SUV**_max_**)**	**1st Week**	**2nd Week**	**3rd Week**	**4th Week**	**P value**
Quantitative positivity	1.5±0.5	1.5±0.5	1.4±0.5	1.3±0.49	0.36
Axilla (level I)	2.0±1.7	1.7±0.98	1.5±0.68	1.5±0.84	0.09
Axilla (level II)	1.7±0.76	1.5±0.7	1.7±0.98	1.8±0.93	0.88
Supra/Infraclavicular	1.9 ±1.2	1.3±0.3	1.8±1.1	1.4±0.37	0.71
Deltoid	1.8±0.92	1.6±0.68	1.2±0.46	1.3±0.56	0.001

## Discussion

 Australia initiated nationwide COVID-19 vaccination in early 2021 using both AstraZeneca and Pfizer vaccines with the goal of immunization coverage greater than 80% of the eligible population before the end of the year. This allowed us to compare ^18^F-FDG PET/CT features and the degree of regional and systemic immune response in patients who were recently vaccinated with either of these two types of vaccines. To the best of our knowledge, this is the first study to compare the local, regional nodal and systemic immune reactions of viral vector and mRNA COVID-19 vaccinations based on ^18^F-FDG PET/CT findings in patients with recent COVID-19 immunization.

 In general, ^18^F-FDG PET/CT was semiquantitatively positive for vaccine-related features in half (50.5%) of the studied population. The ipsilateral axillary (including all levels) and infra- and supraclavicular lymph nodes were significantly larger in size and exhibited higher metabolic activity than the contralateral lymph nodes after both Pfizer and AstraZeneca vaccination; however, the lymph node size did not meet the CT criteria for lymphadenopathy. Although extension of the nodal reaction toward the infra and supraclavicular regions was observed, there was no evidence of vaccine-related nodal reaction within the ipsilateral pulmonary hilar region, and bilateral axillary nodal reaction was observed in less than 2% of patients. Previous data showed increased splenic uptake post vaccination (13); However, our study did not show significant difference in spleen to liver and bone marrow to liver metabolic activity neither between vaccinated and the control group nor between patients who received Pfizer or AstraZeneca vaccines.

 According to our results, the prevalence of ipsilateral hypermetabolic axillary lymph nodes post Pfizer and AstraZeneca vaccination was 39% and 17%, respectively. The achieved prevalence for Pfizer vaccination (39%) is quite similar to the pooled prevalence of 37% (27-47%, 95% CI) reported by a recently published systematic review and meta-analysis. Interestingly, 8 out of 9 included studies in that article assessed ^18^F-FDG PET/CT features in patients who received Pfizer vaccination, which can explain the similar detection rate to our Pfizer group of patients (14). 

 Although the prevalence of hypermetabolic regional lymph nodes was not significantly different between patients received Pfizer and AstraZeneca vaccination (P=0.1); the detection rate of visually positive regional lymph nodes was higher in patients received Pfizer vaccine than that of received AstraZeneca. Additionally, the intensity of nodal metabolic activity and tendency of extension to more remote nodal stations, including level II and III axilla and infra- and supraclavicular lymph nodes, were also higher in patients receiving mRNA vaccines compared with those receiving viral vector vaccines. These findings suggest that patients receiving mRNA vaccines are more vulnerable for having vaccine-related false-positive results on ^18^F-FDG PET studies compared to patients receiving viral vector vaccines. This is the first time this result in being reported.

 Our study showed that while FDG uptake at the injection site was significantly reduced after the first week (and subsequently may not be useful as a clue to recent vaccination), the regional nodal reaction remained constant for 4 weeks post vaccination. There is complete heterogenicity in the reported results in this regard. Some recent studies reported diminished nodal reactions after the first week (15); however, other studies reported the persistence of the nodal reactions at the 10th week post vaccination (16). The persistence of the nodal reaction, as observed in our and the previously mentioned studies, may be explained by the fact that COVID-19 vaccination appears to provoke stronger immune stimulation than prior vaccines, resulting in a longer-lasting immune reaction (17). Given this considerable pitfall, clinical datasheets may need to document vaccination information beyond one month prior to PET study, and nuclear medicine specialists should consider the possibility of vaccine-related nodal reaction even when the vaccination has been performed over one month prior to the PET scan.

 There are certain limitations to this study. First, given the national age-related eligibility criteria for COVID-19 vaccination, the patients’ age was not completely homogenous between patients who received mRNA and viral vector vaccines. Further age-matched studies comparing these two types of immunization against COVID-19 would be of great value. The second drawback involves limiting our studied population to those who had COVID-19 vaccination within the last 30 days prior to PET study. While this criterion resulted in a more homogenous study population, as nodal reactions may persist beyond 4 weeks post vaccination, we could not determine the expected period of nodal reactions post COVID-19 vaccination. Further studies including patients with longer time intervals between vaccination and PET would be desirable to answer this clinical question.

## Conclusion

 Significant local and regional nodal reactions are observed after both viral vector and mRNA COVID-19 vaccination, with a tendency to extend toward the infra- and supraclavicular nodal stations but not to the pulmonary hilum. The prevalence, intensity and extension of the nodal reaction was greater after Pfizer vaccination suggestive of higher possibility of false-positive results on ^18^F-FDG PET/CT studies using mRNA vaccination technology.

## Financial disclosure

 There are no potential conflicts of interest relevant to this article.

## Conflicts of interest

 The authors declare that they have no conflict of interest.

All authors have read and approved the submitted manuscript.
